# 肺腺癌中FAT1的表达及其与免疫细胞浸润关系的研究

**DOI:** 10.3779/j.issn.1009-3419.2024.102.01

**Published:** 2024-02-20

**Authors:** Chen DING, Wenhao ZHAO, Hua HUANG, Yongwen LI, Zhanrui ZHANG, Ruihao ZHANG, Yanan WANG, Di WU, Chen CHEN, Hongyu LIU, Jun CHEN

**Affiliations:** ^1^832003 石河子，石河子大学医学院第一附属医院胸外科（丁晨，张展瑞，陈军）; ^1^Department of Thoracic Surgery, First Affiliated Hospital, School of Medicine, Shihezi University, Shihezi 832003, China; ^2^300052 天津，天津医科大学总医院肺部肿瘤外科（赵文浩，黄华，张芮豪，王亚楠，吴迪，陈军）; ^2^Department of Lung Cancer Surgery, Tianjin Lung Cancer Institute, Tianjin Medical University General Hospital, Tianjin 300052, China; ^3^300052 天津，天天津市肺癌研究所，天津市肺癌转移与肿瘤微环境重点实验室（李永文，陈琛，刘红雨，陈军）; ^3^Tianjin Key Laboratory of Lung Cancer Metastasis and Tumor Microenvironment, Tianjin Lung Cancer Institute, Tianjin Medical University General Hospital, Tianjin 300052, China

**Keywords:** 肺肿瘤, FAT1, 预后, 增殖, 免疫, Lung neoplasms, FAT1, Prognosis, Proliferation, Immune

## Abstract

**背景与目的** 肺癌是癌症相关死亡的主要原因。非小细胞肺癌（non-small cell lung cancer, NSCLC）是肺癌最常见的病理分型，其中又以肺腺癌占比最高。非典型性钙黏蛋白1（FAT atypical cadherin 1, FAT1）是一种受体样蛋白，在肺腺癌中存在高频率突变，其编码的蛋白在细胞黏附、细胞增殖、分化等过程中发挥着重要的作用。本研究旨在研究FAT1在肺腺癌中的表达及与免疫浸润的关系。**方法** 通过下载癌症基因组图谱（The Cancer Genome Atlas, TCGA）和基因型-组织表达（Genotype-Tissue Expression, GTEx）相关数据，获得513例肺腺癌样本、397例癌旁样本的基因表达水平及相关临床信息。分析肺腺癌组织中FAT1基因mRNA表达水平以及FAT1基因的表达与肺腺癌患者的预后关系，通过通路富集分析探索FAT1基因调控的相关信号通路。利用免疫蛋白印迹检测FAT1在肺上皮细胞和不同肺癌细胞系中的表达差异，免疫组织化学染色法被用于检测FAT1在肺癌和癌旁组织中的表达。**结果** 14%的肺腺癌患者中存在FAT1基因突变；TCGA数据库数据显示肺腺癌组织中FAT1 mRNA表达显著高于癌旁组织。Kaplan-Meier分析显示FAT1基因表达较高的肺腺癌患者预后较差。通路富集分析表明FAT1与肿瘤发生发展通路相关，且FAT1的表达和免疫细胞浸润密切相关，免疫组化实验证明FAT1在癌组织的表达显著高于癌旁组织。**结论** 肺腺癌组织中FAT1 mRNA呈高表达，高表达的FAT1 mRNA与肺腺癌患者的不良预后相关。FAT1有可能成为潜在的肺癌诊治靶点。

## Abstract (en)

**Background and objective** Lung cancer is a leading cause of cancer-related deaths. Non-small cell lung cancer (NSCLC) is the most common pathological subtype, with adenocarcinoma being the predominant type. FAT atypical cadherin 1 (FAT1) is a receptor-like protein with a high frequency of mutations in lung adenocarcinoma. The protein encoded by FAT1 plays a crucial role in processes such as cell adhesion, proliferation, and differentiation. This study aims to investigate the expression of FAT1 in lung adenocarcinoma and its relationship with immune infiltration. **Methods** Gene expression levels and relevant clinical information of 513 lung adenocarcinoma samples and 397 adjacent lung samples were obtained through The Cancer Genome Atlas (TCGA) and Genotype-Tissue Expression (GTEx) data. The mRNA expression levels of the FAT1 gene in lung adenocarcinoma tissues were analyzed, along with its association with the prognosis of lung adenocarcinoma patients. Pathway enrichment analysis was conducted to explore the signaling pathways regulated by the FAT1 gene. Immunoblotting was used to detect the differential expression of FAT1 in lung epithelial cells and various lung cancer cell lines, while immunohistochemistry was employed to assess FAT1 expression in lung cancer and adjacent tissues. **Results** FAT1 gene mutations were identified in 14% of lung adenocarcinoma patients. TCGA database data revealed significantly higher FAT1 mRNA expression in lung adenocarcinoma tissues compared to adjacent lung tissues. Kaplan-Meier analysis indicated lower survival rates in lung adenocarcinoma patients with higher FAT gene expression. Pathway enrichment analysis suggested the involvement of FAT1 in tumor development pathways, and its expression was closely associated with immune cell infiltration. Immunohistochemical validation demonstrated significantly higher expression of FAT1 in cancer tissues compared to adjacent lung tissues. **Conclusion** FAT1 mRNA is highly expressed in lung adenocarcinoma tissues, and elevated FAT1 mRNA expression is associated with poor prognosis in lung adenocarcinoma patients. FAT1 may serve as a potential biomarker for lung cancer.

肺癌是全世界癌症相关死亡的主要原因^[1]^。肺癌通常分为两个主要类型：非小细胞肺癌（non-small cell lung cancer, NSCLC）和小细胞肺癌，其中NSCLC约占肺癌患者的85%，分为肺鳞癌（squamous cell carcinoma, SCC）、肺腺癌（lung adenocarcinoma, LUAD）和大细胞肺癌。肺癌的发展通常伴随着肿瘤细胞的侵袭和转移，是肺癌病情严重程度和治疗效果的重要影响因素。肺癌的发生和发展涉及多种分子、细胞和组织的相互作用，因此急需探索潜在的分子靶点，以改善肺癌的治疗。

非典型性钙黏蛋白1（FAT atypical cadherin 1, FAT1），是一种受体样蛋白，属于FAT家族的一部分，FAT家族包括FAT1、FAT2、FAT3和FAT4四种蛋白^[2]^。这一家族的蛋白在多种生物学过程中发挥重要作用，特别是在细胞黏附、细胞增殖、分化和组织发育方面^[3]^。FAT1是一种常见于上皮组织中的跨膜蛋白，其功能包括黏附分子和信号传导受体^[4,5]^。FAT1还参与细胞-细胞黏附的形成，抑制FAT1的表达会导致细胞连接的不稳定性下降，进而破坏细胞的极性^[3]^。既往研究^[6⇓⇓⇓-10]^表明，FAT1在不同类型的肿瘤中发挥着截然不同的作用，一般认为它在宫颈癌、头颈鳞状细胞癌、口腔癌和乳腺癌中发挥抑癌作用，其功能缺失可能促进肿瘤细胞的进展。FAT1功能丧失促进鳞状细胞癌的发生、进展、侵袭性、干性和转移^[11]^。FAT1通过激活Hippo信号传导来抑制NSCLC细胞的肿瘤初始能力^[12]^。相反的是，FAT1可以调控急性髓系白血病的进展，被认为是急性髓系白血病的癌基因^[13]^。FAT1可通过调节胶质母细胞瘤的干细胞相关基因的表达来发挥致癌作用^[14]^。FAT1可促进肝细胞癌的增殖和迁移，并抑制细胞凋亡^[15]^。但是，FAT1对肺癌的影响以及机制尚不清楚，尚需进一步探索。进一步研究FAT1和肺癌之间的关系对于揭示肺癌的生物学机制和潜在治疗靶点至关重要。在课题组之前的一项研究^[16]^中，我们揭示了FAT1突变作为NSCLC生物标志物，有助于识别不太可能从免疫检查点抑制剂中持续临床获益的患者，从而有助于个体化免疫治疗方案的制定。FAT1突变的NSCLC患者肿瘤突变负荷高、免疫应答细胞浸润增加、免疫抑制细胞浸润减少，同时具有更好的免疫原性和免疫治疗效果^[17]^。肿瘤浸润免疫细胞存在于癌症的各个阶段，在调控肿瘤生长中起着重要作用。然而，FAT1与肿瘤免疫细胞浸润丰度的关系还不清楚，急需进一步研究。

此研究旨在通过多数据库生信分析，阐明FAT1在LUAD中的表达、突变等情况，进一步探索其可能影响的通路及其和免疫浸润细胞的关系，通过实验验证FAT1在LUAD细胞中的表达，探讨其成为NSCLC治疗的潜在分子靶点的可能性。

## 1 资料与方法

### 1.1 数据获取

本研究中有关LUAD患者的数据来源于癌症基因组图谱（The Cancer Genome Atlas, TCGA）（https://cancergenome.nih.gov）和基因型-组织表达（Genotype-Tissue Expression, GTEx）数据库，共纳入了910个（513例LUAD样本，397例癌旁样本）样本记录，包括基因表达数据以及相应的临床信息。

### 1.2 基因突变分析

使用cBioportal（https://www.cbioportal.org）数据库探讨LUAD患者FAT1的基因改变^[18]^。为了获得cBioportal中的基因变化，首先从TCGA数据库获得LUAD患者的体细胞突变数据，然后使用GISTIC算法推测出拷贝数变化，以及相对于所有样本的mRNA表达z-score（log RNA Seq V2 RSEM）。最后选取患者集作为所有样本，输入FAT1，得到LUAD患者中FAT1基因突变比例和类型。此外，通过“maftools”包绘制瀑布图来展示FAT1高表达组和低表达组患者的基因表达情况。

### 1.3 差异基因分析和通路富集分析

使用“limma”包^[19]^，对FAT1高表达组与低表达组的基因进行差异分析，采用Log2|FC|>1为截断值，计算两组之间的差异基因。使用“clusterProfiler”包，对这些差异基因进行GO功能分析以及京都基因组百科全书（Kyoto Encyclopedia of Genes and Genomes, KEGG）分析。此外，使用基因集c2.cp.kegg.v7.4.symbols.gmt进行基因富集分析（gene set enrichment analysis, GSEA），探究FAT1潜在参与的通路。

### 1.4 免疫细胞浸润分析

使用ESTIMATE以及单样本GSEA（single-sample GSEA, ssGSEA）计算TCGA LUAD患者中的免疫评分和免疫细胞浸润丰度。

### 1.5 免疫组织化学染色

组织芯片包含91对原发性LUAD组织和相应的癌旁组织，购买于赛维尔生物公司（武汉，中国）。对组织芯片进行脱蜡，脱水，然后用1×EDTA在98 °C处理10 min修复抗原。载玻片在3%过氧化氢溶液中孵育以阻断内源性过氧化物酶。用10%山羊血清封闭后，将组织与抗FAT1（Abcam, ab190242, 1:500）在4 °C下孵育过夜。用PBS冲洗后，载玻片与生物素偶联的二抗孵育，洗涤，并与HRP偶联的链霉亲和素孵育。对载玻片进行复染时，采用苏木素染色法。细胞染色强度分为四个等级，即阴性（得分为0分）、淡黄色（得分为1分）、棕黄色（得分为2分）和棕褐色（得分为3分）。根据阳性细胞百分比进行评分，分为四个等级：≤25%得1分，26%-50%得2分，51%-75%得3分，>75%得4分。最终评分结果是两项评分相乘。为避免偏倚，两位病理学家独立对每个样本进行评分。

### 1.6 细胞培养和蛋白质免疫印迹法

细胞系H1650、H1975、A549、H1299、PC9和BEAS-2B购自中科院典藏生物资源保藏中心。这些细胞系在含有10%胎牛血清的DMEM（诺普赛，PM150210）培养基、37 °C和5%CO_2_的湿润培养箱中培养。蛋白质免疫印迹法：正常肺上皮细胞系和不同LUAD肿瘤细胞系进行培养后，提取细胞总蛋白，BCA法定量各细胞系总蛋白浓度。每个样本取20 μg蛋白，用SDS-PAGE电泳后转膜，含5%脱脂奶粉的TBST封闭1 h，抗FAT1抗体（Abcam, ab190242, 1:1000）、抗GAPDH抗体（Proteintech, 60004-1-Ig, 1:1000）作为一抗，4 °C下一抗孵育过夜，使用TBST洗膜3次，每次10 min，将膜与抗兔/小鼠IgG（Abclone，抗兔：AS014；抗鼠：AS003）二抗在室温下孵育1 h，TBST洗膜3次，每次10 min，在膜上显影条带，并使用Syngene G-Box GeneSnap软件（Syngene, Cambridge, UK）读取显影后条带。

### 1.7 统计分析

所有的分析都是通过R语言4.3.0来进行。Wilcox秩和检验用于比较癌和癌旁组织间的基因表达水平，Kaplan-Meier结合Log-rank检验来比较不同组间的总体生存率，FAT1免疫反应评分与LUAD患者临床病理特征的关系使用卡方检验。P<0.05为差异具有统计学意义。

## 2 结果

### 2.1 基因突变分析

使用cBioportal（https://www.cbioportal.org）数据库，探究FAT1的基因结构和转录变化。通过图1我们发现，14%的患者存在FAT1基因突变，错义突变是FAT1基因的主要改变形式，其次为深度缺失。

**图1:**
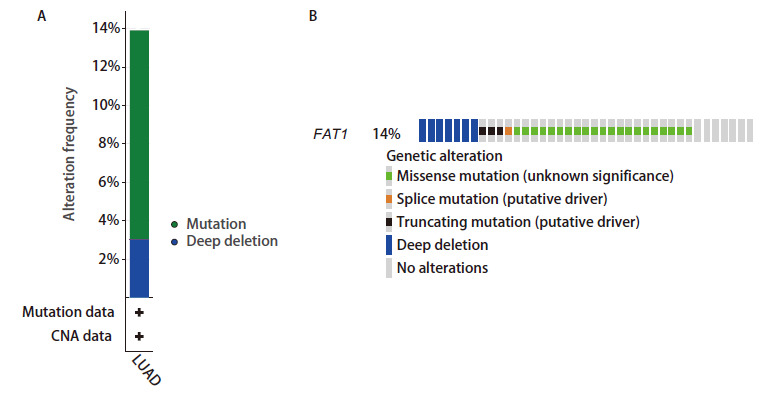
基因突变分析。cBioportal数据库中LUAD患者FAT1基因的突变频率（A）及突变类型（B）。

### 2.2 FAT1在LUAD细胞系和癌组织中表达较高

采用TCGA和GTEx数据库对513例LUAD组织和397例正常肺组织的FAT1 mRNA表达水平进行对比，结果显示，FAT1基因在LUAD组织中的表达水平显著高于正常肺组织，差异具有统计学意义（P<0.0001）（图2A）。进一步验证FAT1 mRNA在同一样本中的癌组织和癌旁组织有着明显的表达差异，癌组织高于癌旁组织（P<0.0001）（图2B）。通过蛋白质免疫印迹法检测LUAD细胞系H1650、H1975、A549、H1299、PC9和正常肺上皮细胞系BEAS-2B中FAT1蛋白的表达，结果表明LUAD细胞系中的FAT1蛋白的表达明显高于正常肺上皮细胞系（P<0.01）（图2C）。对91对LUAD和癌旁组织进行了免疫组织化学染色，表1总结了这个队列患者的临床特征，通过对癌症患者癌组织和正常癌旁组织的FAT1基因表达水平进行比较分析，发现FAT1基因在癌组织中的表达显著上调（P<0.0001）（图2D）。在高评分组中患者年龄>60岁的比例更高（P<0.001），然而在T分期和临床分期中，患者比例并无明显差异。

**表1:** FAT1免疫反应评分与LUAD患者临床病理特征的关系

Clinical characteristics	FAT1		Total (n=91)	P
	Low (n=44)	High (n=47)		
Age (yr)				<0.01
<60	35 (79.55%)	4 (8.51%)	39 (42.86%)	
≥60	9 (20.45%)	43 (91.49%)	52 (57.14%)	
Gender				0.99
Female	30 (68.18%)	33 (70.21%)	63 (69.23%)	
Male	14 (31.82%)	14 (29.79%)	28 (30.77%)	
T stage				0.56
T1	29 (65.91%)	26 (55.32%)	55 (60.44%)	
T2	4 (9.09%)	9 (19.15%)	13 (14.29%)	
T3	5 (11.36%)	6 (12.77%)	11 (12.09%)	
T4	6 (13.64%)	6 (12.77%)	12 (13.19%)	
Clinical stage				0.69
I	27 (61.36%)	29 (61.70%)	56 (61.54%)	
II	7 (15.91%)	9 (19.15%)	16 (17.58%)	
III	10 (22.73%)	8 (17.02%)	18 (19.78%)	
IV	0 (0.00%)	1 (2.13%)	1 (1.10%)	

**图2:**
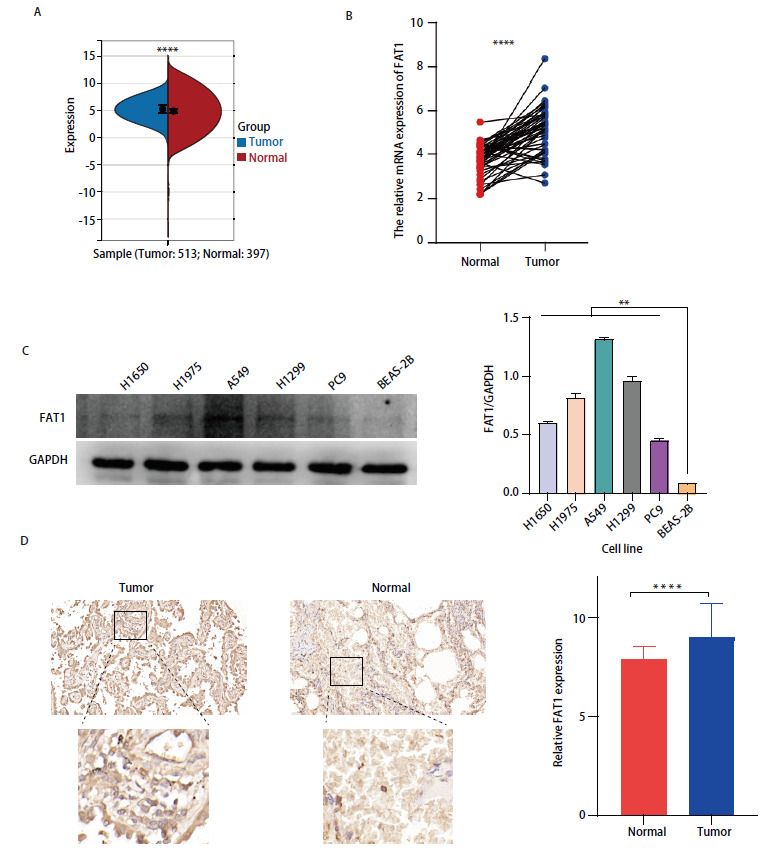
FAT1在肺腺癌细胞系和癌组织中表达较高。A：TCGA数据库中FAT1在癌和癌旁组织中的表达情况；B：FAT1在同一癌和癌旁组织中的表达情况；C：蛋白质免疫印迹法显示正常肺上皮细胞和肺腺癌细胞系中FAT1蛋白的表达差异；D：免疫组织化学染色显示在肺腺癌组织芯片中，FAT1在癌组织的表达显著高于癌旁组织（放大倍数：上，×100；下，×200）。

### 2.3 FAT1与LUAD患者的预后不良相关

以FAT1表达量的中位数为截断值，将TCGA 数据库中LUAD患者分为高、低表达两组，Kaplan-Meier plotter在线分析绘制LUAD患者的总体生存期曲线。结果显示，TCGA数据库中FAT1的表达量与LUAD患者的总体生存期显著相关，高表达FAT1患者的总体生存期显著低于低表达患者（P<0.05）（图3）。

**图3:**
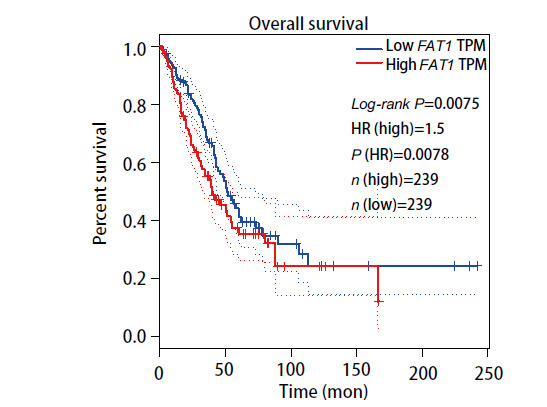
FAT1高、低表达组之间的Kaplan-Meier生存分析

### 2.4 FAT1的通路富集分析

为了探索FAT1可能参与的功能通路，对FAT1高表达和低表达组进行了差异分析，得到1452个差异基因（图4A）。为了探究这些基因的潜在生物学功能，对其进行了GO以及KEGG分析，GO分析结果显示主要富集在抗菌免疫应答、突触后膜电位的调节、雌激素代谢过程、消化等通路（图4B）。KEGG结果显示这些差异基因主要与中性粒细胞胞外杀菌网形成、系统性红斑狼疮、酒精中毒等通路相关（图4C）。GSEA结果显示在FAT1低表达组，这些差异基因主要与移植物抗宿主病、核糖体、产生IgA的肠道免疫网络、抗原加工和呈递、金黄色葡萄球菌感染等通路相关联（图4D）。在FAT1高表达组，这些差异基因主要与尼古丁成瘾、系统性红斑狼疮、酒精中毒、中性粒细胞胞外杀菌网形成、细胞外基质受体相互作用等通路相关联（图4E）。

**图4:**
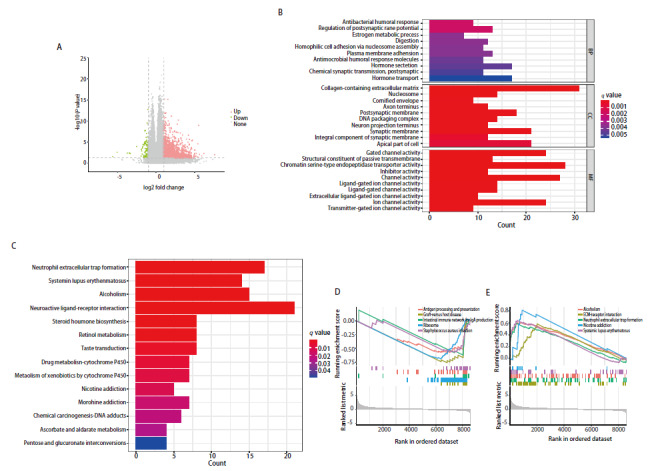
FAT1的通路富集分析。A：火山图展示了癌和癌旁组织之间的差异基因；B：直方图展示了差异基因的GO分析结果；C：柱状图展示了差异基因的KEGG分析结果；D：差异基因分别在FAT1低表达组和高表达组的GSEA分析结果。

### 2.5 FAT1与免疫细胞浸润和免疫检查点的表达密切相关

肿瘤微环境对肿瘤的发生发展至关重要。我们进一步评估了20余种类型免疫细胞在FAT1高、低表达两组之间的浸润丰度差异，结果显示，活化B细胞、活化CD8^+^ T细胞、骨髓来源抑制性细胞、单核细胞在FAT1高表达组浸润丰度更低，而中性粒细胞、II型辅助性T细胞在FAT1高表达组浸润丰度更高（P<0.05）（图5A）。我们对免疫检查点相关基因在FAT1高、低表达组之间的表达水平进行了比较，结果显示CD276、TNFRSF14、TNFRSF18、LAG3、PDCD1、TNFSF4的表达水平在两组之间具有明显差异（图5B），提示FAT1的表达与LUAD的发生发展及治疗相关。

**图5:**
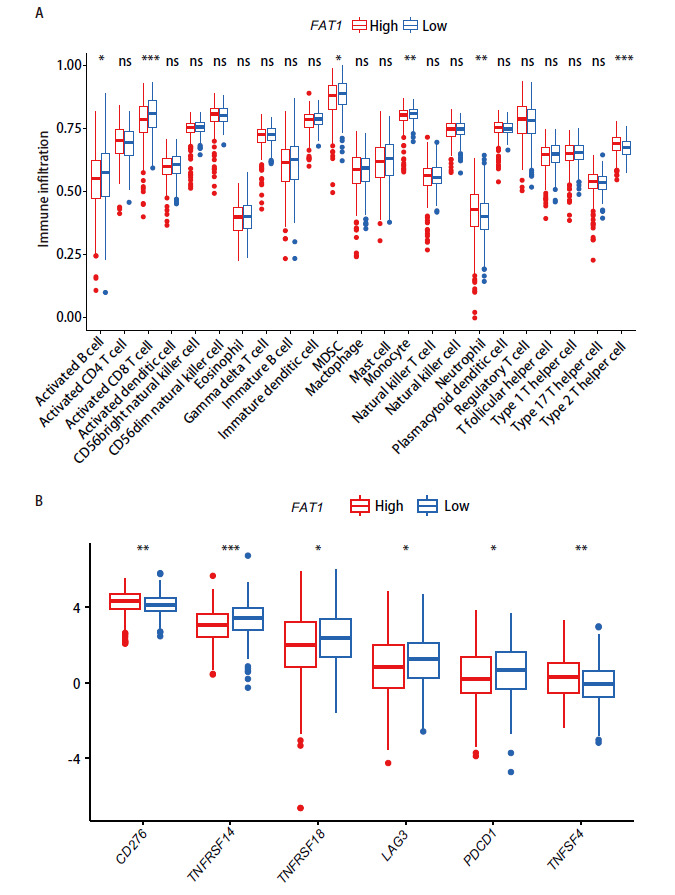
FAT1与免疫细胞浸润和免疫检查点的表达密切相关。A：箱式图展示了免疫细胞在FAT1高、低表达组的表达水平；B：箱式图展示了免疫检查点相关基因在FAT1高、低表达组的表达水平。

## 3 讨论

肺癌的发病率和致死率在全球范围内居高不下，对公共卫生构成严重威胁^[20]^。FAT1是一种受体样蛋白，它在肺癌等多种癌症中引起了广泛的关注。FAT1基因编码的蛋白在细胞黏附、细胞增殖等过程中发挥着重要的作用^[21]^。目前的研究证明FAT1在多种癌症中发挥癌基因或抑癌基因的作用。因此，阐明FAT1和肺癌之间的关系具有重要意义。

在这项研究中，我们发现FAT1在LUAD组织和癌旁组织之间表达存在显著差异。这表明FAT1的表达水平可能与LUAD进展有关。这一发现对深入了解FAT1的生物学功能以及其在LUAD发展中的作用方面具有重要意义。Kaplan-Meier生存分析结果显示，FAT1低表达患者具有更好的预后，这一发现为FAT1作为潜在的肺癌治疗靶点提供了依据。通过cBioportal数据库的分析，研究发现FAT1基因在LUAD组织中存在频繁突变，其中错义突变是主要的变化形式，提示FAT1基因可能在肺癌发生发展中扮演重要角色，错义突变可能对其功能产生负面影响。FAT1在细胞间黏附和间质的调控中发挥作用，影响肿瘤细胞与周围环境的相互作用。这可能对肿瘤微环境的结构和组织产生影响，影响免疫细胞的浸润。FAT1突变可能是对免疫治疗没有持久临床获益的NSCLC患者的预测性生物标志物^[16]^。分析表明FAT1的表达水平与程序性死亡配体1（programmed death ligand 1, PD-L1）的表达呈正相关，这可能与肿瘤对免疫治疗的响应有关^[22]^。了解FAT1与免疫治疗效果之间的关系有助于预测患者的治疗反应。深入研究FAT1在肿瘤微环境免疫浸润中的机制，有望为开发新的肿瘤治疗策略提供重要的信息。

这项研究探讨了FAT1基因在肺癌中的作用，包括其表达水平、基因变异以及免疫调控。然而，需要注意的是，尽管我们的研究从多个方面探究了FAT1在LUAD中的表达和功能，但它仍存在一些局限性。首先，这是一项基于数据库和体外实验的研究，尚未涉及到体内动物实验或临床试验。因此，虽然我们发现了FAT1与不良预后的关联，但其具体机制尚需进一步研究，尤其需要深入了解FAT1在体内动物模型中的作用。其次，由于数据仅涉及FAT1基因的表达，我们并未深入探讨与FAT1相关的分子信号通路。最后，该研究局限于LUAD，不同类型的肺癌和其他癌症类型中FAT1的作用仍需要更广泛的研究。总之，这些发现不仅有助于深化对肺癌发展机制的理解，还为开发针对FAT1的治疗方法提供了新的线索，具有潜在的临床应用前景。本研究为肺癌的治疗和生存率预测提供了信息，有望为未来的癌症研究和治疗提供有力支持。

**Competing interests**

The authors declare that they have no competing interests.

**Author contributions**

Liu HY and Chen J designed the research studies. Ding C, Zhao WH, Huang H and Zhang ZR performed the experiments. Chen C, Wang YN, Wu D, Zhang RH and Li YW analyzed data. Ding C and Huang H wrote the manuscript. Liu HY provided financial support. Liu HY and Chen J supervised the project. All authors made comments on the manuscript.
